# Postoperative Complications Result in Poor Oncological Outcomes: What Is the Evidence?

**DOI:** 10.3390/curroncol31080346

**Published:** 2024-08-15

**Authors:** Anjana Wajekar, Sohan Lal Solanki, Juan Cata, Vijaya Gottumukkala

**Affiliations:** 1 Department of Anesthesiology, Critical Care and Pain, Advanced Centre for Treatment Education and Research in Cancer, Tata Memorial Centre, Homi Bhabha National Institute, Mumbai 410210, India; anjanawajekar@gmail.com; 2 Department of Anesthesiology, Critical Care and Pain, Tata Memorial Hospital, Homi Bhabha National Institute, Mumbai 400012, India; 3 Department of Anesthesiology and Perioperative Medicine, MD Anderson Cancer Center, Houston, TX 77030, USA; jcata@mdanderson.org (J.C.); vgottumukkala@mdanderson.org (V.G.)

**Keywords:** postoperative complications, cancer outcomes, overall survival, recurrence-free survival, initiation of adjuvant therapies

## Abstract

The majority of patients with solid tumors undergo a curative resection of their tumor burden. However, the reported rate of postoperative complications varies widely, ranging from 10% to 70%. This narrative review aims to determine the impact of postoperative complications on recurrence and overall survival rates following elective cancer surgeries, thereby providing valuable insights into perioperative cancer care. A systematic electronic search of published studies and meta-analyses from January 2000 to August 2023 was conducted to examine the effect of postoperative complications on long-term survival after cancer surgeries. This comprehensive search identified fifty-one eligible studies and nine meta-analyses for review. Recurrence-free survival (RFS) and overall survival (OS) rates were extracted from the selected studies. Additionally, other oncological outcomes, such as recurrence and cancer-specific survival rates, were noted when RFS and OS were not reported as primary outcomes. Pooled hazard ratios and 95% confidence intervals were recorded from the meta-analyses, ensuring the robustness of the data. The analysis revealed that long-term cancer outcomes progressively worsen, from patients with no postoperative complications to those with minor postoperative complications (Clavien–Dindo grade ≤ II) and further to those with major postoperative complications (Clavien–Dindo grade III–IV), irrespective of cancer type. This study underscores the detrimental effect of postoperative complications on long-term oncological outcomes, particularly after thoracoabdominal surgeries. Importantly, we found a significant gap in the data regarding postoperative complications in surface and soft tissue surgical procedures, highlighting the need for further research in this area.

## 1. Introduction

Worldwide, the incidence of cancer is on the rise. Approximately 60% of patients with solid tumors require surgery as a part of cancer management [1]. Postoperative complications are defined as any deviations from the normal postoperative course, which includes asymptomatic complications but excludes cancer sequelae and recurrence [2]. Postoperative complications after major curative cancer surgery are common. The rate of these complications varies from 10 to 70%, depending on factors such as cancer staging, preoperative cancer therapies (neoadjuvant), patients’ physiological reserve and functional capacity following neoadjuvant therapies, coexisting medical comorbidities, the type and complexity of the surgery, and the extent of resection [1,3] There is evidence regarding the short-term outcomes of these postoperative complications, including perioperative morbidity and mortality, increased length of hospital stay, and financial burden [1,2,3]. A pioneering study by Khuri et al. [4] in 2005 analyzed data from 105,951 patients (from the National Surgical Quality Improvement Program database in the United States) who underwent eight different types of surgical procedures (both oncological and non-oncological). The study reported that 30-day postoperative complications reduced the median patient survival by 69% [5]. Five-year mortality in patients with any 30-day postoperative complications was 57.6%, compared to 39.5% in patients with no complications. The study also found that mortality rates varied with the type and severity of complications, with five-year mortality after perioperative myocardial infarction as high as 73%, compared to 58% in patients with urinary tract infections.

Regarding cancer, some believe that surgery may stimulate cancer growth and dissemination through mechanisms such as the release of circulating tumor cells, the disruption of stromal tissue, and induced neuro-inflammatory signaling, resulting in an endocrine–metabolic stress response [5,6,7,8]. Other researchers have implicated cancer-mediated immune suppression in the formation of micrometastases and further seeding [9]. The use of neo-adjuvant chemotherapy has also shown to have an immune-modulatory role, potentially impacting both perioperative complications and cancer recurrence [10]. Given the inflammatory–immune responses and alterations in the systemic milieu following major postoperative complications, there has been a growing interest in exploring the association between postoperative complications and long-term outcomes in a wide spectrum of cancers, including colorectal, gastric, and breast cancers. Furthermore, the severity of postoperative complications may also affect the resumption of timely postoperative adjuvant therapy, delaying the return to intended oncological treatment and potentially worsening long-term oncological outcomes [2]. This article reviews the impact of postoperative complications after major curative oncological surgery on long-term oncological outcomes, including recurrence-free survival (RFS) and overall survival (OS).

## 2. Materials and Methods

The primary objective of this narrative review was to evaluate the published literature regarding RFS and OS following postoperative complications after potentially curative oncological surgery. In this review, disease-free survival is reported under RFS. Other long-term oncological outcomes, such as cancer-specific survival, overall recurrence rates, and local or distant recurrence rates, are documented only if the included studies did not report RFS or OS. The secondary objectives were to record the incidence and nature of the most frequent postoperative complications, including surgical site infections, anastomotic leaks, bowel perforation, renal dysfunction, cardiovascular and respiratory complications, bleeding, and others. Additionally, the grading and severity of these complications were assessed.

### 2.1. Selection Criteria, Search Strategies, and Data Collection

An electronic literature search on PubMed, Embase, Scopus, and Google Scholar was conducted for peer-reviewed English language articles using the terms “postoperative complications”, “postsurgical complications”, “long-term cancer outcomes”, “long-term oncological outcome”, “recurrence-free survival”, “overall survival”, “disease-free survival”, “local recurrence”, “cancer-specific survival”, “distant recurrence”, “anastomotic leak”, “wound complications”, and “septic complications” in different combinations. All randomized controlled trials, non-randomized controlled trials, cohort studies, and observational studies published between January 2000 and August 2023 addressing postoperative complications and the specified survival outcomes were included in the review. Abstracts without full-text access, duplicates, and non-English language texts were excluded, as well as those with animal model studies, case reports, studies with incomplete text, and conference proceedings.

### 2.2. Data Extraction and Synthesis

All articles were independently evaluated by two researchers who reported all collected data in an Excel 2021 (Microsoft, Redwood, MS, USA) spreadsheet designed for the purposes of this study. The collected information included the year of publication, place of study, type of study, inclusive period of study, aim of the study, inclusion and exclusion criteria, definitions of postoperative complications and postoperative mortality, number of patients, and OS and/or RFS rates.

Due to the heterogeneity in the published and included studies, data synthesis was conducted using a qualitative approach (narrative synthesis) to summarize and interpret different parameters. The postoperative complications and other outcome parameters were calculated by averaging reported percentages or means and standard deviations, or by converting medians with ranges or interquartile ranges to approximate means and standard deviations using established formulas [5]. These converted values were combined using an inverse-variance weighted method to derive the final estimates.

### 2.3. Literature Search Results

**Figure 1:**
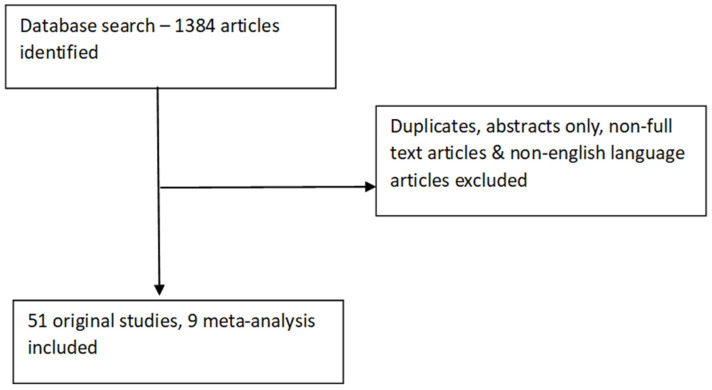
Flowchart of study selection.

A total of 1384 articles were identified after the initial literature search (Figure 1). The initial review, conducted by two authors, involved screening the article titles for relevance. The full-text assessment identified 51 original articles and 9 meta-analyses focusing on postoperative complications and long-term cancer outcomes for inclusion in the final review. The disease site distribution of the included studies was as follows: colorectal (n = 19), urological (n = 6), colorectal liver metastasis (n = 5), gastrointestinal (n = 2), hepato-pancreatico-biliary (n = 4), peritoneal (n = 3), thoracic (n = 3), breast (n = 4), soft tissue sarcoma (n = 2), and head and neck (n = 2) malignancies [1,3,6,11,12,13,14,15,16,17,18,19,20,21,22,23,24,25,26,27,28,29,30,31,32,33,34,35,36,37,38,39,40,41,42,43,44,45,46,47,48,49,50,51,52,53,54,55,56,57,58]. Table 1 contains demographic details and the Table 2 contains the details of the postoperative complications and cancer outcomes data of the various studies included in the review. Table 3 includes all the meta-analysis data [59,60,61,62,63,64,65,66,67].

## 3. Results

While most studies have reported 30-day outcomes, postoperative complications have also been reported in 90-day outcomes [1,11,13,31,33,45,46,53]. These complications are further graded using different classification systems: the Clavien–Dindo (CD) classification, the Comprehensive Complication Index (CCI), and the National Cancer Institute Common Terminology Criteria for Adverse Events (CTCAE) ver. 5.0, which are among the most common [68,69,70].

### 3.1. Classification Systems for Grading POSTOPERATIVE COMPLICATIONS

The CD classification has been commonly used to grade surgical complications based on the level of intervention required to achieve resolution. Due to its simplicity, uniform reporting across a wide range of surgeries, and low inter-rater variability, it is a popular classification system. However, a few drawbacks of the CD classification system include excluding intraoperative complications and reporting only the highest-grade complication, thereby excluding any “lesser” complications. The CCI summarizes all the postsurgical complications, calculating a cumulative burden of morbidity on a continuous scale ranging from 0 to 100. Despite its comprehensive approach, the CCI has not yet found widespread application due to its complex calculations [7,8].

For the purpose of this review, we have combined the surgical procedures for gastrointestinal, colorectal, hepato-pancreatico-biliary, peritoneal, urological, and thoracic malignancies under the umbrella term of “thoracoabdominal surgeries”. All other procedures, such as those for breast, extremity soft tissue sarcoma, and head and neck malignancies, are grouped under “surface and soft tissue surgeries”. Postoperative complications are commonly identified based on anatomy (cardiovascular, respiratory, or renal) or their mechanism (infection/sepsis, hematoma/effusions, etc.).

### 3.2. Reported Postoperative Complications Grading Systems

The challenge of heterogeneity in the classification systems used to grade complications in published literature is significant. While the CD classification system is the predominant system, being utilized in 23 out of the selected 51 studies, other systems are also in use. Four studies have employed the CCI, and two have used the CTCAE version 3.0. Additionally, a study from Potkrajcic et al. [55] on soft tissue sarcoma utilized a major wound complication classification. Furthermore, four studies used both the CD and CCI systems to grade postoperative complications. Among these studies, two focused on patients with colorectal liver metastasis and peritoneal malignancies, respectively, and reported that the CCI was a better prognostic indicator of immediate postoperative morbidity, re-admission, and long-term survival. Despite the seemingly better correlation of cancer outcomes with the CCI classification grade, the CD system remains more commonly used. It is observed that long-term cancer outcomes progressively worsen from patients with no postoperative complications to those with minor and then major postoperative complications, irrespective of the cancer type. Given the heterogeneity in reporting methodologies and the differences in outcomes related to the reporting system, it is critically important to standardize the reporting system of postoperative complications following cancer surgery. This standardization would facilitate more consistent and comparable research findings, ultimately contributing to improved patient care and outcomes.

### 3.3. Overall Impact of Postoperative Complications on Cancer Outcomes

There is extensive literature on the incidence and severity of postoperative complications after surgical procedures in patients with cancer, as well as the numerous factors that predict the development of these complications. Multivariate analysis has identified higher age, patient comorbidities, surgical technique (open vs. minimally invasive), surgical duration and complexity, and intraoperative blood loss as some of the most common predictors of postoperative complications across a range of cancers and, consequently, their long-term outcomes [7,8,25,37,50].

#### 3.3.1. Thoracoabdominal Surgery

The presence of even a single postoperative complication led to worse RFS in one study, whereas others reported worsening RFS only when patients suffered both surgical and medical postoperative complications [8,14,43]. After colorectal cancer surgery, multiple postoperative complications led to shorter OS [13]. Several studies have reported poor long-term oncological outcomes in patients undergoing colorectal resections with or without hyperthermic intraperitoneal chemotherapy, followed by major postoperative complications [26,39,40]. Although multi-visceral resections for colon cancer independently increased the rates of both postoperative complications and local recurrence, there was no direct association between postoperative complications and 5-year RFS or OS [3]. Fukami et al. [32] reported that postoperative complications were independently associated with OS only after repeat hepatectomies for colorectal hepatic metastasis, but not primary hepatectomies. Meanwhile, Yin et al. [66], in their meta-analysis, reported that postoperative complications were strongly correlated with poorer long-term outcomes. Wang et al. [64], in their meta-analysis, concluded that postoperative complications, especially infectious and anastomotic leaks, correlated with worse outcomes in stage II and III gastric carcinomas, but its effect in stage I gastric carcinoma is indeterminate. The negative effect of the severity of postoperative complications on 2-year RFS and OS persists even after oncologic lung resections, from patients with no postoperative complications to those with minor postoperative complications (CD grade ≤ II) and major postoperative complications (CD grade III–IV) [48]. Several meta-analyses in non-metastatic colorectal cancers, gastric cancer, and hepatocellular cancers found that postoperative complications have a significant harmful impact on RFS (a cumulative hazard ratio of 1.35 [95% CI 1.29–1.40]) and OS (a cumulative hazard ratio of 1.46 [95% CI 1.37–1.55]) [59,60,61,62,63,64,65,66,67].

#### 3.3.2. Surface and Soft Tissue Surgeries

Only one study by Broecker et al. [56] reported that postoperative complications following truncal and extremity soft tissue sarcomas led to significantly reduced RFS and OS compared to the group with no postoperative complications. We found a significant gap in the data on postoperative complications in surface and soft tissue surgical procedures, highlighting the need for further research in this area.

### 3.4. Subgroup Analysis of Types of Postoperative Complications and Their Impact on Cancer Outcomes

#### 3.4.1. Anastomotic Leak

An anastomotic leak is a significant surgical complication following thoracoabdominal surgery, with incidence rates varying from 3% to 40% [18,65]. Anastomotic leaks are associated with longer surgical duration, increased intraoperative bleeding, higher conversion rates from minimally invasive to open surgery, and prolonged postoperative hospital stays [18,65]. They often require surgical, radiological, or endoscopic intervention. Out of 19 studies on colorectal cancer patients, 13 identified anastomotic leak as one of the most frequent postoperative complications. Most studies have reported that anastomotic leaks after colorectal cancer surgeries are associated with significantly lower RFS and/or OS [17,18,24,25,28,29,65,67]. The cumulative 5-year RFS (from 6 studies) and OS (from 5 studies) are (mean (SD)) 67.37% (11.85%) and 71.8% (15.06%), respectively. Ptok et al. [25] reported that only anastomotic leaks requiring surgical treatment were linked to poor 5-year RFS. Only two single-center studies reported that anastomotic leaks did not result in reduced RFS or OS in colorectal cancer [14,21]. Biliary leaks were identified as common postoperative complications after hepatectomy in only two out of eight studies, which found postoperative complications to be an independent predictor of reduced RFS and OS [30,36]. Neenan et al. [34] reported no association between major postoperative complications, particularly pancreatic fistula, and RFS, OS, or local recurrence after pancreaticoduodenectomy.

#### 3.4.2. Wound Complications

The incidence of wound complications following cancer surgery ranges from 6% to 30% [18,52,54]. Postoperative wound complications have a multifactorial pathophysiology. Wound infections are often the most common source of nosocomial infections in these patients. These complications can lead to both local and systemic manifestations, worsening not only short-term outcomes, such as increased length of hospital stay, but also potentially impacting long-term cancer outcomes.

##### Wound-Related Complications in Thoracoabdominal Surgeries

Wound-related complications were identified as common and major issues in five studies on colorectal surgeries and three studies on hepato-pancreatico-biliary surgeries. Sprenger et al. [18] reported a significant decline in 10-year OS (45.7%) and an increase in local recurrence (17.3%) in patients with wound complications after rectal surgery. Although wound complications (4.5%) were the third most frequent postoperative issue following radical colorectal resection, they were not associated with poorer cancer outcomes [26]. A study by Kube et al. [23] found a higher incidence of wound complications in patients with anastomotic leaks after colon surgery and significantly poorer RFS and OS in patients with major postoperative complications. Another study in the post-hepatectomy cohort found wound complications (including infections and dehiscence) to be the most common postoperative complications, but these were not significantly related to either RFS or OS [37].

##### Wound-Related Complications in Surface and Soft Tissue Surgeries

Three out of four studies on breast cancer surgeries reported that wound complications did not lead to poorer oncological outcomes [51,52,53]. However, one older single-center study involving breast cancer patients, with data collected before 2002, reported increased rates of systemic recurrence [54]. Potkrajcic et al. [55] found that postoperative major wound complications occurred most frequently in patients with diabetes mellitus, but these complications did not affect long-term oncological outcomes after soft tissue sarcoma excision. In a study of total laryngectomy patients, wound complications were identified as the most common postoperative issue and were found to be an independent predictor of decreased long-term cancer outcomes [57].

#### 3.4.3. Other Infectious Complications

In surface and soft tissue surgeries, only one study in total laryngectomy patients mentioned pneumonia (5.8%) as the second most frequently occurring postoperative complication. It concluded that postoperative complications lead to significantly poorer RFS and OS. A study on colonic oncological surgeries reported an incidence of 2.85% infective complications (including surgical infectious complications) and 2.53% non-infectious complications, which were graded as CD grade ≥ III [19]. The study found that the overall recurrence rate was similar in groups with and without complications; however, both local anastomotic site and peritoneal recurrence were more common in stage III colorectal cancer patients with major complications. Septic complications following hepatectomy and colorectal resections have been shown to significantly affect 1-, 3-, 5-, and 10-year RFS and/or OS in several studies [26,31,35,37].

#### 3.4.4. Non-Infectious Complications

After rectal surgeries, compared to cases without postoperative complications, regardless of grade, infectious complications and intestinal dysmotility complications were associated with worse RFS, while cardiopulmonary and thromboembolic complications were linked to reduced OS. Postoperative renal dysfunction was associated with both worse RFS and worse OS [13]. Among non-infectious complications post-hepatectomy, Chok et al. [37] identified liver failure, cardiac complications, renal failure, pulmonary complications, and postoperative hemorrhage, in that order, as significantly associated with reduced OS. One study reported worse long-term survival after nephrectomy for individual complications such as acute renal failure, cardiac complications, and septic or neurologic complications [47]. Law et al. [26] found that cardiopulmonary complications significantly worsened 5-year OS but did not affect overall recurrence rates. The surface and soft tissue surgery studies included in this review did not report the rates and impact of non-infectious complications.

## 4. Discussion

Given the increasing global incidence of cancer and the growing number of patients with solid tumors requiring curative resections to control their tumor burden, it is crucial to understand the perioperative factors that can be optimized to improve cancer outcomes. In this literature review, we found that postoperative complications lead to poorer long-term oncological outcomes after thoracoabdominal surgeries. Specifically, categorizing the complications and their effects on outcomes revealed that anastomotic leaks significantly contribute to poorer oncological outcomes in colorectal surgeries [17,18,24,25,28,29,65,67]. There is a lack of data on postoperative complications in non-coelomic cancer surgeries. Long-term oncological outcomes progressively worsen, from patients with no postoperative complications to those with minor and then major postoperative complications, regardless of cancer type.

When evaluating perioperative factors on oncological outcomes, two additional considerations are the role of neoadjuvant therapies in postoperative complications and the impact of postoperative complications on delays in initiating planned adjuvant therapies.

### 4.1. Role of Neoadjuvant Chemotherapy on Postoperative Complications

Neoadjuvant chemotherapy is incorporated into cancer treatment regimens to induce tumor shrinkage, improve resectability, and enhance survival [10]. Chemotherapy-induced leukocytopenia and neutropenia are well-documented effects. Additionally, chemotherapy has been reported to have immunomodulatory effects, promoting lymphocyte activation and reducing the production of inhibitory immune cells [10]. However, the impact of neoadjuvant chemotherapy on postoperative complications and, consequently, on long-term cancer outcomes, has yielded contrasting results.

Takeuchi et al. [49] reported that administering neoadjuvant chemotherapy mitigated the negative impact of postoperative complications on long-term cancer outcomes after esophagectomy. In contrast, Wu et al. [41] found that postoperative complications worsened RFS in gastric cancer patients treated with neoadjuvant chemotherapy. Sprenger et al. [18] reported worse 10-year OS (51%) in patients with anastomotic leaks after rectal surgery compared to those without anastomotic leaks, regardless of perioperative chemotherapy use. The nuanced effects of neoadjuvant therapies on postoperative complications and cancer-specific outcomes warrant further exploration.

### 4.2. Postoperative Complications and Delay in Initiation of Adjuvant Treatment

Another mechanism speculated for poor long-term outcomes after postoperative complications may be the delay in receiving adjuvant therapy. Several studies have reported that the occurrence of postoperative complications leads to an increased length of ICU or hospital stays, higher re-surgery rates, and readmission within 90 days [1,6,12,17,23,26,37,53,56]. Whether this results in a delay in the onset of adjuvant treatment after surgery and consequently worsens cancer outcomes is a subject of much discussion. A few studies on patients with colorectal liver metastasis, breast cancer, and soft tissue sarcomas have reported no delay in the initiation of adjuvant therapy between groups with and without postoperative complications, and even between groups with minor versus major complications [6,33,54,56]. Similarly, some studies on colorectal and breast cancer patients have shown no association between the occurrence of postoperative complications and long-term cancer outcomes when adjusted for the delay in starting adjuvant chemotherapy [13,14,53]. However, a study of total laryngectomy patients found that both postoperative complications and delays in adjuvant therapy were independent predictors of decreased disease-free survival (DFS) and OS [57]. Krarup et al. [22] reported that patients with stage III colon carcinoma who experienced anastomotic leaks were less likely to receive adjuvant chemotherapy, or its initiation was significantly delayed (16 days, 95% CI: 12–20 days) compared to patients without anastomotic leaks. This delay in receiving adjuvant chemotherapy led to a significant reduction in OS but not in distant recurrence. The effects of postoperative complications on the initiation of adjuvant therapy and outcomes related to RFS and OS are intriguing and evolving areas of research. Further studies are needed in the context of specific diseases and cancer stages to reach a conclusive understanding.

## 5. Limitations of the Current Literature on Postoperative Complications and Oncological Outcomes

As previously reported, contaminated surgical sites are a risk factor for an increased likelihood of postoperative complications. Among our cohort of studies included for analysis, those on colorectal surgery patients constituted the largest group. The data from these studies are heterogeneous in their reporting of both postoperative complications and long-term oncological outcomes. Studies have used various terms, such as local, distant, or overall recurrence rates, cancer-specific survival, etc. This complexity is further compounded by subgroup analyses based on different postoperative complication grading systems, RAS gene mutations, and surgical techniques (open, laparoscopic, conversion). Some studies focus on a single surgical complication and its effect on cancer outcomes, while others include all postoperative complications. This heterogeneity makes it challenging to synthesize meaningful data.

## 6. Future Research

Further research is needed to identify procedure-specific risk factors for the development of postoperative complications and to understand the mechanisms through which different complications impact long-term cancer outcomes. Additional areas for research include surgical techniques, the role of perioperative chemo-radiation therapies, and the impact of enhanced recovery pathways on the risk of postoperative complications. Finally, optimizing patients in the preoperative period—especially concerning frailty and prehabilitation—and employing continuous vital sign monitoring technologies, as well as utilizing machine learning for early risk prediction and implementing rapid rescue measures, may improve long-term oncological outcomes.

## 7. Conclusions

Most of the studies included in this review focus on thoracoabdominal surgeries, where postoperative complications are linked to poorer long-term oncological outcomes. Specifically, anastomotic leaks contribute to worse outcomes in colorectal surgeries. There is a lack of sufficient data on postoperative complications in surface and soft tissue cancer surgeries. For these types of surgeries, wound complications are reported as the most common postoperative issues and are associated with higher cancer recurrence rates. Among the studies on thoracoabdominal cancer surgeries, comparisons of postoperative complication grading systems found that the high CCI was a better predictor of complications than the CD classification. Long-term cancer outcomes progressively worsen from patients with no postoperative complications to those with minor and major complications, regardless of cancer type.

## Figures and Tables

**Table 1:** Demographics of original articles.

Studies	Publication Year	Data Duration	Type of Study	No of Institutes (Country)	Cancer Diagnosis	Surgery	Type or Surgery	Sample Size	Age	Age (Statistical Terms)	Female (%)	NACT (%)
Colorectal												
Koedam et al. [11]	2022	1997–2003, 2004–2010	RC	Multicentre (8 countries)	Colorectal CA	Resection anastomosis	Open, Lap	1076 (COLOR), 764 (COLOR II)	NS	NS	NS	NS
Bao et al. [12]	2022	2009–2016	PC	Multicentre (Italy)	Rectal CA	Low anterior resection	open, Lap	311	63.6 ± 10.9	Mean (SD)	40.5	81
Gamboa et al. [13]	2021	2007–2017	RC	Multicentre (USA)	Rectal CA	Proctectomy (LAR/APR)	Open, Lap, robotic	1136	59 (51–67)	Median (IQR)	39	76
Fransgaard et al. [14]	2021	2010–2015	RC	One (Denmark)	Colorectal CA	NS	NS	4083	NS	NS	45.16	NS
Wasmann et al. [3]	2020	2000–2014	RC	Multicentre (Belgium and Denmark)	Colon CA	Multi-visceral resections	Open, Lap, conversion	130	68	Mean	47	NS
Oh et al. [15]	2020	2010	RC	One (Republic of Korea)	Colorectal CA	NS	NS	310	60.5 (32–85)	Mean (Range)	33.8	NS
Miyamoto et al. [16]	2020	2005–2017	PC	One (Japan)	Colorectal CA	NS	NS	673	69 (19–95)	Median (range)	39	NS
Furnée et al. [17]	2019	2011	RC	Multicentric (The Netherlands)	Rectal CA	Low anterior resection with primary anastomosis	Open, Lap, conversion	746	57.45	Mean	NS	NS
Sprenger et al. [18]	2018	1995–2002	RC	Multicentre (Germany)	Rectal CA	Resection anastomosis	NS	799	62 (30–77)	Median (range)	31.4	50.8
Cienfuegos et al. [19]	2018	2000–2014	RC	Multicentre (Spain)	Colorectal CA	NS	Open, Lap, conversion	950	66.2	Mean	39.05	NS
Park et al. [20]	2016	2005–2012	RC	One (Republic of Korea)	Rectal CA	Low anterior resection	Open, LAP	686	62.2 (28–89)	Mean (range)	38.63	NS
Espin et al. [21]	2015	2006–2008	RC	Multicentre (Spain)	Rectal CA	Low anterior resection	NS	1153	NS	NS	35.21	53.69
Krarup et al. [22]	2014	2001–2008	RC	Multicentre (Denmark)	Colon CA	Resection anastomosis without ostomy	Open, Lap	8589	72 (23–98)	Median (range)	52.41	NS
Kube et al. [23]	2010	2000–2004	RC	Multicentre (Germany)	Colon CA	Colonic resection with anastomosis	NS	844	NS	NS	NS	NS
Marra et al. [24]	2009	1991–2004	RC	One (Switzerland)	Colon CA	Resection anastomosis	NS	440	68.6 (22–99)	Mean (range)	39.55	NS
Ptok et al. [25]	2007	2000–2001	RC	Multicentre (Germany)	Rectal CA	Resection	NS	303	66 (32–92)	Median (range)	32.01	6.6
Law et al. [26]	2007	1996–2004	RC	One (China)	Colorectal CA	Radical resection	Open, Lap	1657	70 (24–94)	Median (range)	43.09	NS
McArdle et al. [27]	2005	1991–1994	RC	Multicentre (Scotland)	Colorectal CA	Resection	NS	2235	NS	NS	50.25	NS
Walker et al. [28]	2004	1971–1999	RC	Multicentre (Australia)	Colorectal CA	Resection	NS	1722	NS	NS	5.11	NS
Bell et al. [29]	2003	1971–1991	RC	One (Australia)	Rectal CA	Anterior Resection	NS	403	67 (31–94)	Median (range)	29.8	NS
Colorectal Liver metastasis (CRLM)												
Wang et al. [30]	2022	2007–2018	RC	One (China)	CRLM	Hepatectomy	Open, lap	751	58 (51.0–64.0)	Median (IQR)	35.4	65.8
Fernández-Moreno et al. [31]	2020	2000–2016	RC	One (Spain)	CRLM	Hepatectomy	Open, lap	254	63.66 (±10.98)	Mean (SD)	39.8	NS
Yamashita et al. [6]	2017	2008–2014	RC	One (USA)	CRLM	Hepatectomy	NS	575	56 (18–88)	Median (range)	40.7	86
Fukami et al. [32]	2016	1994–2015	RC	One (Japan)	CRLM	Hepatectomy	Open, Lap	282	64 (10)	Mean (SD)	38.65	6
Mavros et al. [33]	2013	2000–2009	RC	One (USA)	CRLM	Hepatectomy (and Radiofrequency ablation)	NS	251	58 (51–68)	Median (IQR)	34.7	76.9
Hepato-pancreatico-biliary												
Neeman et al. [34]	2019	2008–2016	PC	One (Israel)	Pancreatic CA	Pancreaticoduodenectomy	NS	148	66 (41–85)	Median (range)	40	NS
Ma et al. [35]	2018	1991–2013	RC	One (Hong Kong)	Cholangio CA	Hepatectomy	NS	107	61(25–79)	Median (range)	45.79	4.67
Harimoto et al. [36]	2015	2004–2012	RC	Three (Japan)	Hepatocellular CA	Hepatectomy	NS	966	68	Mean	31.6	NS
Chok et al. [37]	2009	1989–2004	RC	One (Hong Kong)	Hepatocellular CA	Hepatectomy	Open	863	54 (12.5)	Mean (SD)	18.42	NS
Peritoneal												
Choudry et al. [38]	2018	2001–2016	RC	One (USA)	Peritoneal CA	CRS HIPEC	NS	1296	55.8 (47.1–63.8)	Median (IQR)	NS	NS
Schneider et al. [39]	2017	2009–2014	RC	One (Switzerland)	Peritoneal CA/metastasis	CRS HIPEC	Open, Lap	113	52 (43–59)	Median (IQR)	50.44	NS
Baratti et al. [40]	2014	2004–2012	RC	Two (Italy)	Peritoneal metastasis	CRS HIPEC	NS	101	59.4(10.4)	Mean (SD)	60.39	NS
Gastro-intestinal												
Wu et al. [41]	2019	2006–2016	RC	One (China)	Gastric CA	Gastrectomy (subtotal and total)	NS	500	NS	NS	36.4	NS
Climent et al. [42]	2015	1990–2009	RC	One (Spain)	Gastric CA	Gastric resection	NS	271	69 (7)	Mean (SD)	41	0
Urology												
Notarfrancesco et al. [1]	2023	2010–2020	RC	Two (Switzerland)	Metastatic germ cell CA testis	Post-chemo RPLND	Open, lap, robotic	136	31.3 (17.3–69.8)	Median (range)	NS	100
Leonard et al. [43]	2020	2008–2016	RC	One (France)	Prostate CA in Renal transplant patients	Radical prostatectomy	Robotic	27	63.3 [43–73]	Mean (range)	100	NS
Muto et al. [44]	2017	2012–2016	RC	One (Japan)	Bladder CA	Radical Cystectomy	Robotic Open	49	68.55	Mean	20.4	36.73
Cusano et al. [45]	2016	2003–2013	RC	One (USA)	Bladder CA	Cystectomy	Open or robotic	213	67 (10.4)	Mean (SD)	21.12	27.7
Nguyen et al. [46]	2014	2001–2013	RC	One (Switzerland)	Bladder CA	Robot assisted radical cystectomy	Robotic	61	83 (80–94)	Median (range)	11	NS
Tan et al. [47]	2012	1995–2005	RC	Multicentric (USA)	Renal Cell CA	Partial/radical nephrectomy	Open, Lap	12,618	NS	NS	42.13	NS
Thoracic												
Barea et al. [48]	2021	2012–2014	RC	One (Spain)	Lung cancer	Lung resection	NS	146	55–78	Range	36.3	NS
Takeuchi et al. [49]	2019	2000–2017	RC	One (Japan)	Esophageal CA	Esophagectomy	NS	431	64 (34–85)	Median (range)	14.1	NS
Kinjo et al. [50]	2012	2002–2010	RC	One (Japan)	Esophageal CA	Esophagectomy	Thoracoscopic, open	185	63.4	Mean	15	NS
Breast												
Machiels et al. [51]	2020	2007–2018	RC	One (Belgium)	Breast CA	Breast Conservation Surgery + radiotherapy	NS	763	NS	NS	100	NS
Teoh et al. [52]	2020	2011–2018	RC	One (Malaysia)	Breast CA	Mastectomy with/out reconstruction	NS	421	53.16 (±10.75)	Mean (SD)	100	NS
Mousa et al. [53]	2017	2009–2016	RC	One (Israel)	Breast CA	Alloplastic breast reconstruction	Open	186	48.9 (21–77)	Median (range)	100	13
Murthy et al. [54]	2007	1994–2001	RC	One (UK)	Breast CA	Mastectomy, breast conservation	Open	1065	58 (22–98)	Median (range)	100	NS
Sarcoma												
Potkrajcic et al. [55]	2022	2011–2017	RC	One (Germany)	Soft tissue sarcoma	Excision	Open	74	59.6 (18–87)	Mean (range)	36	58.1
Broecker et al. [56]	2017	2000–2015	RC	One (USA)	Soft tissue sarcoma	Excision	Open	546	55 (12–93)	Median (range)	46	12
Head and Neck												
Boukovalas et al. [57]	2020	2008–2013	RC	One (USA)	Laryngeal CA	Total laryngectomy	Open	362	63.6	Mean	18.8	39.5
Milliet et al. [58]	2018	2000–2015	RC	One (France)	Laryngeal or hypopharyngeal CA	Total pharyngo-laryngectomy	Open	245	66.4 (35–90)	Mean (range)	11	48

NS—Not specified, RC—Retrospective cohort, PC—Prospective cohort, CA—carcinoma, CRS HIPEC—Cytoreduction surgery with hyperthemic intraperitoneal chemotherapy, Lap—Laparoscopy, RPLND—Retroperitoneal lymph node dissection.

**Table 2:** Postoperative complications and long-term oncological outcomes in the original studies.

Studies	Morbidity (%)	Morbidity (Grade ≥ III-%)	Classification	Commonest Surgical Complications			Follow-Up (Months)	RFS (%)	RFS (Years)	Other Oncological Outcomes	OS (%)	OS (Years)	POSTOPERATIVE COMPLICATIONS Correlation to RFS (Yes/No)	POSTOPERATIVE COMPLICATIONS Correlation to OS (Yes/No)
				First (%)	Second (%)	Third (%)	Median (Range)							
Colorectal														
Koedam et al. [11]	6.1		NS	AL (NS)	NS	NS	60	Colon (50.9), rectal (53.6)	5	NA	Colon (58.5), Rectal (69.3)	5	Rectal-Y, Colon-N	Rectal-Y, Colon-N
Bao et al. [12]	30.2	12.9	CD	AL (20.3)	NS	NS	69.5 (31.9) m(SD)	80.7, 75.1, and 63.5	3, 5, and 10	NA	89.2, 85.3, 70.2	3, 5 and 10	N	N
Gamboa et al. [13]	46	32	CD	Infectious (20)	Intestinal dysmotility (19)	Renal (9)	31 (IQR 13–54)	48	5	NA	64	5	Y	Y
Fransgaard et al. [14]	67.8	NS	NS	NS	NS	NS	NS	NS	NS	Hazard ratios of RFS and OS calculated for delay in adjuvant therapy	NS	NS	N	N
Wasmann et al. [3]	35	NS	NS	NS	NS	NS	56	NS	NS	26% local recurrence	NS	NS	NS	NS
Oh et al. [15]	NS	37.4	extended CD	Wound complications (31.6%)	Ileus (19.7)	AL (11.9)	72.2 (0.2–113.6)	81.5	5	NS	NS	NS	N	NS
Miyamoto et al. [16]	12.6	NS	CD	SSI (4)	AL (4)	Bowel obstruction (3)	41.5	74	5	NS	NS	NS	Y	Y
Furnée et al. [17]	NS	NS	NS	AL (14.2)	NS	NS	48	NS	NS	RFS and OS calculated for groups with/without AL and with surgical technique	NS	NS	Y	Y
Sprenger et al. [18]	NS	NS	NS	Wound complications (14.39)	AL (12.76)	NS	NS	63.2	10	15.5% local recurrence	46.6	10	Y	Y
Cienfuegos et al. [19]	NS	5.3	CD	NS	NS	NS	84.8	68.8, 32.1	5,10	NS	48.3, 32.2	5, 10	Y	Y
Park et al. [20]	25.51	16.53	CD	AL (7.9)	Intestinal obstruction (4.66)	Anastomotic stricture	43.6 (IQR 26–58)	77.7	5	7.8% local recurrence	89.2	5	Y	N
Espin et al. [21]	NS	NS	NS	AL (9.4)	NS	NS	60	NS	NS	19.4% overall recurrence	77.5	5	N	N
Krarup et al. [22]	NS	NS	NS	AL (5)	NS	NS	63.6 (IQR 43.2–87.6)	NS	NS	14.9% distant recurrence	NS	NS	Y	Y
Kube et al. [23]	NS	NS	NS	AL (100)	Wound infection (2.9)	Wound dehiscence (1.2)	23	63	5	NS	51	5	Y	Y
Marra et al. [24]	NS	NS	NS	Pneumonia (6.36)	UTI (6.13)	Wound infection (2.95)	63.1 (0.3–193.6)	NS	NS	5.7% local recurrence, 11.3% distant recurrence	33.3 (with AL), 63.7 (without AL)	5	N	Y
Ptok et al. [25]	NS	NS	NS	AL (100)	NS	NS	40	70.9	5	NS	NS	NS	Y	NS
Law et al. [26]	27.3	NS	NS	Pulmonary (5.7)	Cardiac (5.2)	Wound (4.5)	45.3	NS	NS	29.1% overall recurrence, 74.7% 5-year CSS	64.9	5	Y	Y
McArdle et al. [27]	NS	NS	NS	AL (3.85)	NS	NS	NS	NS	NS	OS 42% in patients with AL, 55.1% in patients without AL. CSS 61% with Al, 32% without AL	NS	5	Y	Y
Walker et al. [28]	NS	NS	NS	AL (5.1)	NS	NS	129.6 (60–276)	NS	NA	OS 44.3% with AL, 64% without AL	NS	5	Y	Y
Bell et al. [29]	NS	NS	NS	AL (100)	NS	NS	NA	NS	NA	11.7% 5 year local recurrence	NS	NA	Y	NS
Colorectal Liver metastasis														
Wang et al. [30]	28.8	11.6 (CD), 19(CCI)	CD and CCI	Infection	Biliary leak	Ascites	30 (3–154)	NS	NA	Hazard ratios calculated for different POSTOPERATIVE COMPLICATIONS grading systems	NS	NA	Y	Y
Fernández-Moreno et al. [31]	38.1	NS	CD for surgical and all by CCI	NS	NS	NS	40.5 (76–99)	31	5	NA	62	5	Y	Y
Yamashita et al. [6]	100	15	CCI	NS	NS	NS	37 (6.1–96)	NS	3	RFS calculated for both POSTOPERATIVE COMPLICATIONS group (high and low CCI) and with and without RAS mutation	NS	NS	Y	NS
Fukami et al. [32]	17.4	8.9	CD	Wound infection (6)	Colorectal leak (2.8)	Intra-abdominal abscess (2.1)	48 (12–192)	NS	NS	79.5% and 57.4% 3- and 5-year OS after repeat hepatectomy	39.5, 23.6	3, 5	NS	Y
Mavros et al. [33]	21.91	5.6	CD	Pulmonary (8)	Gastrointestinal (7.2)	Cardiac (3.6)	33.6 (15.6–62.4)	19.5	5	NS	41.9	5	Y	Y
Hepato-pancreato-biliary														
Neenan et al. [34]	NS	19.59	CD	Pancreatic fistula (8.1)	Major wound complications (4.73)	Hemorrhage (2.7)	22 (2–102)	15.5	3	NS	20	5	N	N
Ma et al. [35]	32.7	20.6	CD	Pleural effusion (15.88)	Pneumonia (8.4)	Liver or renal failure (6.5 each)	24 (3.19–276.27)	27	3	NS	27	5	Y	Y
Harimoto et al. [36]	NS	17.1	CD	Bile leak (3.4)	Wound infection (3)	Abdominal abscess (2.9)	40.8	23.7	5	NS	48.6	5	Y	Y
Chok et al. [37]	33.4	NS	NS	Wound complications (9.7)	Pulmonary (8)	Liver failure (5)	35.6	NS	NS	NS	41.5, 26.6	5, 10	N	Y
Peritoneal														
Choudry et al. [38]	66	24	CD, CCI	NS	NS	NS	55	14	5	NS	39	5	NS	NS
Schneider et al. [39]	41.7	10.6	CD	NS	NS	NS	28	NS	3	26, 38, 96% 3-year RFS colorectal, high grade appendiceal, and low grade appendiceal CA	NS	NS	Y	NS
Baratti et al. [40]	NS	23.8	NCI-CTCAE	AL/perforation (6.93)	Hematological toxicity (5.94)	Abdominal abscess (4.95)	44.9 (24.1–65.7)	14.3	5	NS	11.7	5	Y	Y
Gastro-intestinal														
Wu et al. [41]	26.5	19.1	NS	Surgical (20.5)	General (19.1)	Infectious (14.8)	25.7 (12.3–48)	53.6	3	NS	63.4	3	Y	N
Climent et al. [42]	59.8	10	CD, CCI	Intraabdominal sepsis (13.6)	Respiratory sepsis (7)	CLABSI (5.2)	149.9 (140.1–159.9)	NS	NS	NS	55.8, 48.1	5, 10	N	N
Urology														
Notarfrancesco et al. [1]	30.9	9.55	CD	Ileus	Circulatory	Pulmonary	37.2 (0.1–142.1)	20.6	5	41.3% local recurrence, 58.6% distant recurrence or tumor marker positive	90.45	5	N	N
Leonard et al. [43]	29.6	7.4	CD	NS	NS	NS	34.9	NS	NS	RFS 26.9 months in transplant patients	NS	NS	NS	NS
Muto et al. [44]	NS	NS	NS	Pyelonephritis (10.2)	Neobladder stenosis (4.08)	Rectal injury (4.08)	21.75 (7–32)	NS	NS	mean: RFS, 37.4 months; OS, 40.2 months	NS	NS	NS	NS
Cusano et al. [45]	NS	19.24	CD	Gastrointestinal (22.07)	Vascular (14.55)	Infection (13.14)	NS	NS	NS	22.3% vs. 34.8% recurrence in robotic vs. open surgery	NS	NS	NS	NS
Nguyen et al. [46]	44	14.75	CD	Infectious (38)	Gastrointestinal	Cardiac	36	73	2	NS	61	2	NS	NS
Tan et al. [47]	37	NS	NS	Gastrointestinal (12.4)	Pulmonary failure(7.5)	Genitourinary(6.4)	32 (1–132)	NS	NS	NS	59.9	5	NS	Y
Thoracic														
Barea et al. [48]	46.6	11.6	CD	Atelectasis with bronchoscopy	Bleeding	Empyema	48	NS	NS	NS	64.7	2	Y	Y
Takeuchi et al. [49]	71.3	NS	CD	RLN palsy (26.4)	Pneumonia (19.7)	AL (15.8)	NS	59	3	NS	69.5	3	NS	NS
Kinjo et al. [50]	58.38	NS	NCI-CTCAE	Pulmonary (28.65)	Recurrent Laryngeal Nerve palsy (16.76)	Anastomotic leak (12.97)	33.33 (3–95)	NS	2	71.6%, 57.7%, and 58.3% RFS, in thoracoscopic-lap group, thoracoscopic and open groups	NS	NS	N	NS
Breast														
Machiels et al. [51]	3.5	NS	NS	Hematoma (2.6)	Wound infection (0.66)	Wound dehiscence (0.26)	62.2 (0.5–135)	95.1	5	NA	97.2	5	Y	Y
Teoh et al. [52]	NS	NS	NS	Seroma(13)	SSI (7.8)	Bleeding/Hematoma (4.7)	44	NS	NS	8.3% local and 12.8% Distant recurrence	NS	NS	NS	NS
Mousa et al. [53]	45	NS	NS	Dehiscence or infectious (16)	Skin necrosis (10)	Hematoma (4)	40.28	NS	NS	(4%)Local or regional, (7%)Distant recurrence, (3%) both	NS	NS	N	NS
Murthy et al. [54]	NS	NS	NS	Wound complications (9)	NS	NS	NS	82.2	5	NA	NS	NS	Y	NS
Soft tissue Sarcoma														
Potkrajcic et al. [55]	NS	NS	MWC scoring	Wound complications	NS	NS	57.96 (74.4)	77.4	5	NS	91.9	5	NS	NS
Broecker et al. [56]	29	16	CD	NS	NS	NS	37 (0–185)	40	5	35% recurrence (39% local and 61% distant)	NS	NS	Y	NS
Head and Neck														
Boukovalas et al. [57]	37.6	25.4	CD	Wound complications (22.1)	Pneumonia (5.8)	Total flap loss (2.3)	21.1 (0.2–132.9)	NS	NA	29% local, 29% distant recurrence	NS	NS	Y	Y
Milliet et al. [58]	NS	NS	NS	Salivary fistula (31.43)	NS	NS	NS	31	5	NS	36	5	NS	NS

NS—Not specified, AL—Anastomotic leak, RFS—Recurrence free survival, OS—Overall survival, CSS—Cancer specific survival, CD—Clavien–Dindo classification, CCI—Comprehensive Complication Index, MWC—Major Wound Complications, NCI-CTCAE—National cancer institute common terminology criteria for adverse effect version 3.0, CLABSI—Central line associated bloodstream infection, POSTOPERATIVE COMPLICATIONS—Postoperative complications, SSI—Surgical site infection.

**Table 3:** Postoperative complications and long-term oncological outcomes in the meta-analysis.

Studies	Data Duration	Publication Year	Cancer Diagnosis	Studies	Sample Size	RFS [Pooled HR (95% CI)]	OS [Pooled HR (95% CI)]	Other Oncological Outcomes	RFS/OS (Year)	POSTOPERATIVE COMPLICATIONS Impact on RFS	POSTOPERATIVE COMPLICATIONS Impact on OS
Mualla et al. [60]	NS	2021	Non-metastatic Colorectal CA	16 (1 RCT, 3 PC, 12 RC)	37,192	1.41 (1.11–1.80)	1.36 (1.15–1.61)	NS	NS	Y	Y
Kong et al. [61]	2000–2019	2021	Hepatocellular Carcinoma	37 (RC)	14,096	1.25 (1.16–1.35)	1.39 (1.28–1.5)	NS	5	Y	Y
Chen et al. [62]	2009–2020	2021	Gastric CA	32 (RC)	32,067	1.49 (1.33–1.67)	NS	NS	NS	Y	Y
Li et al. [59]	1998–2018	2020	Gastric CA	64 (49 RC, 15 PC)	46,198	1.66 (1.13–2.44)	1.58 (1.37–1.82)	NS	NS	Y	Y
Dorcaratto et al. [63]	NS	2019	Colorectal Liver metastasis	41	12,817	1.38 (1.27–1.49)	1.43 [1.3–1.57]	NS	5	Y	Y
Wang et al. [64]	1986–2015	2019	Gastric CA	16 (RC)	12,065	1.28 (1.10–1.49)	1.40 (1.06–1.84)	NS	NS	Y	Y
Lu et al. [65]	1982–2015	2016	Rectal cancer	11 (5 PC, 6 RC)	13,655	NS	NS	1.61 (1.25–2.09) Local recurrence	NS	Y	Y
Yin et al. [66]	1991–2007	2015	Colorectal Liver metastasis	5 (4 PC, 1 RC)	2370	1.37 (1.23–1.53); 1.34 (1.17–1.53)	1.52 (1.27–1.83); 1.36 (1.18–1.58)	NS	5; 10	Y	Y
Mirnezami et al. [67]	1965–2009	2011	Colorectal CA	21 (1 RCT, 13 non RCT, 7 RC)	21,902	NS	1.64 (1.4–1.91)	2.05 (1.51–2.8) Local recurrence	NS	Y	Y

NS—Not specified, CA—Carcinoma, RCT—Randomized controlled trial, RC—Retrospective cohort, PC—Prospective cohort, RFS—Recurrence-free survival, OS—Overall survival, HR—Hazard ratio, CI—Confidence interval, Y—Yes.
